# Supplementary material for: Dental Emergencies in an Italian Pediatric Hospital during the COVID-19 Pandemic
Source: Healthcare (Basel). 2022 Mar 15;10(3):537. doi: 10.3390/healthcare10030537 (PMC8949433; doi:10.3390/healthcare10030537)
Supplement: Supplementary file 1 [file healthcare-10-00537-s001.zip › healthcare-1628654-supplementary.pdf]

healthcare

MDPI

Supplementary Materials

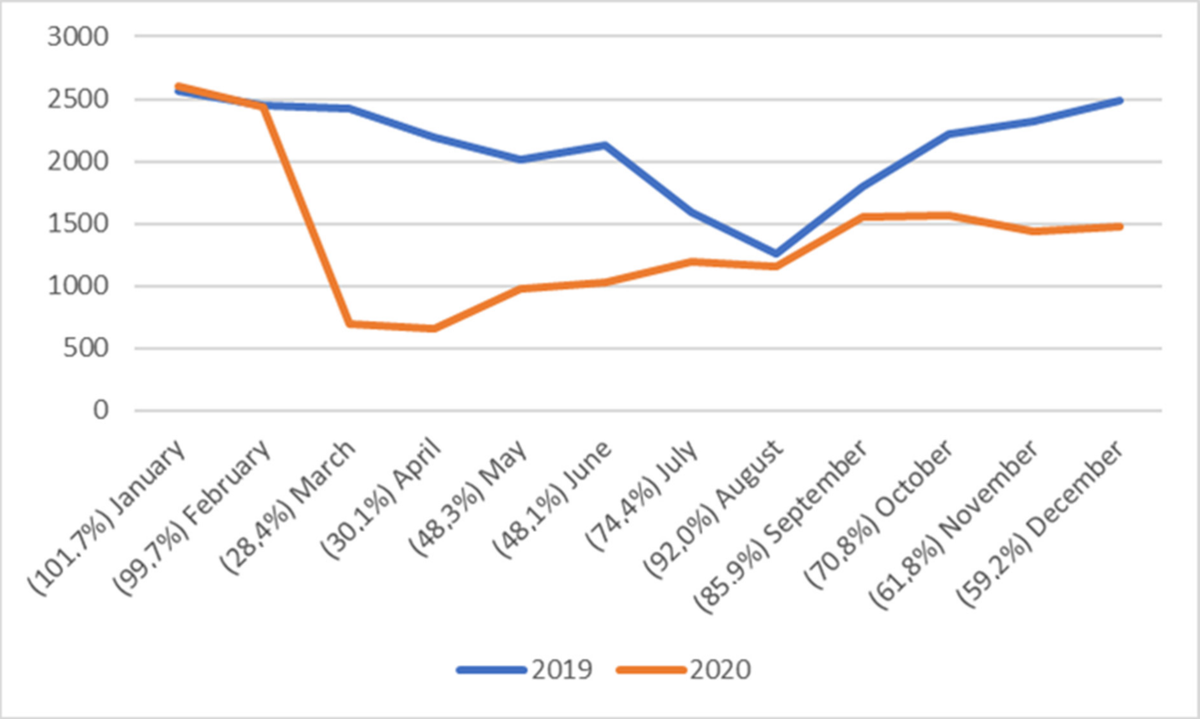

Figure S1. Represents the number of overall ER visits in 2019 (blue) and 2020 (red). In brackets, the proportion of 2020 over 2019.

Table S1. Hospital emergencies. Number and proportion (on the total per year) of the different general reasons for visiting the ER in 2019 and 2020.

Hospital emergencies	2019	2020
Respiratory and ear, nose and throat conditions	7815 (30.7%)	3242 (19.4%)
Trauma/injuries	3452 (13.6)	3007 (18.0%)
Gastrointestinal infections	2407 (9.5%)	831 (5.0%)
Aspecific pain/headache	1751 (6.9%)	1096 (6.5%)
Fever/seizures	653 (2.6%)	585 (3.5%)
Paediatric exanthematous diseases	562 (2.2%)	218 (1.3%)
Allergies/rush	338 (1.3%)	123 (0.7%)
Other	8457 (33.2%)	7648 (45.7%)
Total	25436 (100%)	16750 (100%)

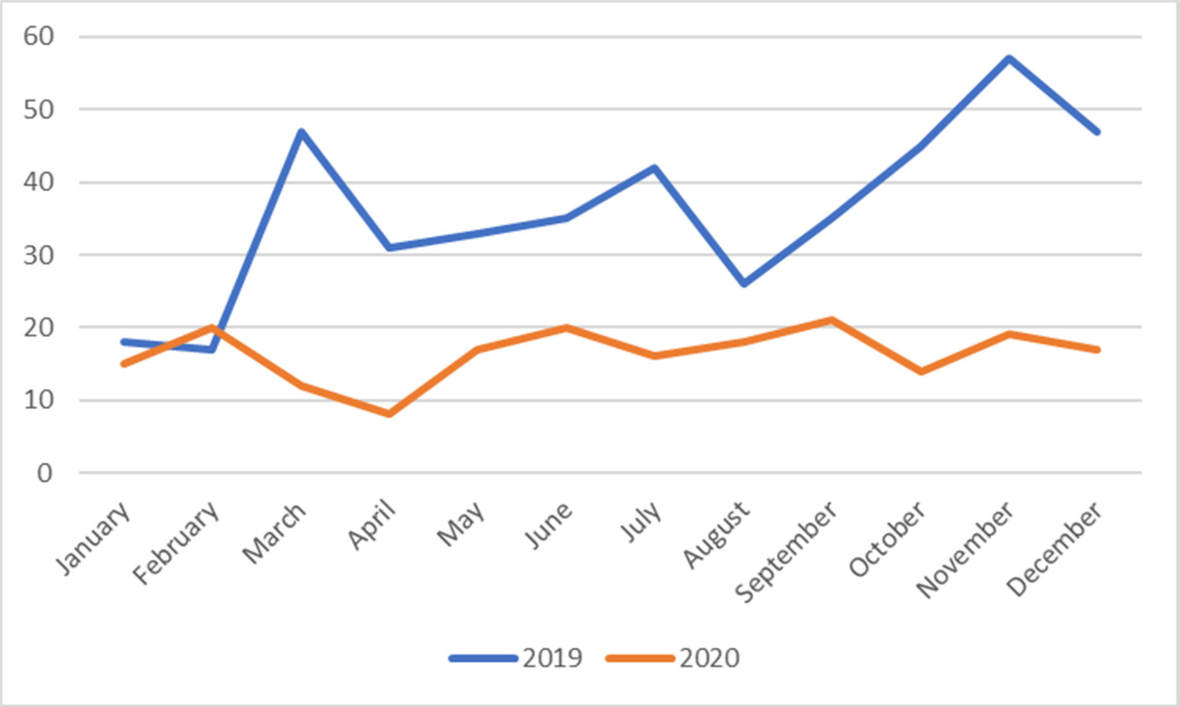

Figure S2. represents the trend for oral ER visits in 2019 (blue) and 2020 (red).

Table S2. Oral emergencies. Reasons for visiting the ER for oral complaints in 2019 and 2020 (number and proportion on the total per year).

Oral emergencies	2019	2020
Stomatitis/aphthous stomatitis	156 (36.0%)	30 (15.2%)
Trauma	76 (17.6%)	21 (10.7%)
Infections/abscesses	32 (7.4%)	19 (9.6%)
Painful caries	32 (7.4%)	10 (5.1%)
Other	137 (31.6%)	117 (59.4%)
Total	433 (100%)	197 (100%)
